# Catheter securement systems: comparison of two investigational devices to a sutureless securement device, a securement dressing, and sutures in a pig model

**DOI:** 10.1186/s40635-015-0060-3

**Published:** 2015-08-27

**Authors:** Laura F. Rutledge, Daniel P. DeCabooter, Shelley-Ann H. Walters, Stéphanie F. Bernatchez

**Affiliations:** Critical & Chronic Care Solutions Division, 3M Company, 3M Center Bldg 270-3A-04, St. Paul, MN 55144-1000 USA

**Keywords:** Catheters, Central venous catheters, Peripheral venous catheterization, Models, Animal, Dressings, Intravenous administration, Vascular access devices, Catheter securement

## Abstract

**Background:**

Catheter securement is critical for the success of infusion therapy and to prevent complications. Our purpose was to compare the strength of catheter securement achieved with two investigational adhesive securement devices to two securement products and also to sutures using an in vivo animal model.

**Methods:**

Twenty-five live pigs were prepared for aseptic abdominal surgery. Four central venous catheters were inserted per animal into the epigastric veins and secured with four of the five securement systems studied, following a balanced incomplete randomized block design. A peak axial pull force test method was used to measure the force required to dislodge the catheter 1 cm from the insertion site and/or cause failure of the device and/or dressing. This pull test was done 10 min after device application, per constraints of the animal model. Comparison analysis was carried out using a mixed effects model with pig, sample, and sample location as factors. Non-inferiority testing was carried out using 95 % confidence intervals with a margin of 4.52 N or 1 lb (454 g). Tukey’s method was used to adjust for multiple pairwise comparisons.

**Results:**

Results showed that the two investigational devices displayed the highest mean peak axial pull forces (40–41 N) and were significantly better than sutures (28 N, *p* < 0.0001) and the securement dressing (17 N, *p* < 0.0001) and non-inferior to the securement device (37 N) in this test. The securement device required a higher mean peak axial pull force than sutures (*p* = 0.0007) and the securement dressing (*p* < 0.0001) for failure to occur. Finally, there was also a statistical difference between sutures and the securement dressing, with sutures requiring a higher mean peak axial pull force for catheter dislodgement than the securement dressing (*p* < 0.0001).

**Conclusion:**

The two investigational devices appear to be a promising alternative for catheter securement, superior to sutures and the securement dressing, and non-inferior to the securement device.

## Background

Catheter securement is essential to the success of short-term (<10 days), non-tunneled central venous catheter (CVC) infusion therapy. These short-term CVCs provide an avenue for various therapies, namely the delivery of medications and solutions that cannot be safely infused into the peripheral circulation, fluid resuscitation, repeated blood product replacement, short-term hemodialysis, and the monitoring of hemodynamic status [1]. If these catheters are not effectively secured to the skin, they can become displaced, causing a wide range of complications and safety issues [2, 3]. The various factors affecting catheter securement have been recently reviewed [4].

Catheter securement can be accomplished using several different methods. The most frequently used method of securing short-term CVCs to the skin is silk or nylon sutures [2, 5, 6]. However, the use of sutures is associated with needlestick or sharp injuries to the health-care worker, as well as patient discomfort and the consequences related to disrupting the patient’s skin barrier [7]. To address these issues, manufactured catheter stabilization devices have entered the market in the last decade and are gaining in acceptance to provide securement [8]. Since 2006, various guidelines recommend the use of such securement devices [1, 8–10]. A recent literature review also found that securement with adhesive systems is more effective than suturing to reduce blood stream infections [11]. Yet, changing practice in health care can take a long time and physicians are likely to continue using sutures for securing CVCs until a larger body of evidence exists for an effective alternative.

To generate evidence comparing sutureless catheter securement methods to sutures, a pig model was used so catheters could be inserted into a dynamic system while avoiding the ethical issues associated with the use of a human model. A peak axial pull force test method was used to determine the relative strength of each securement system in preventing dislodgement under a constant axially directed force. The objective of this study was to explore the catheter securement performance of two novel sutureless systems compared to commercially available sutureless catheter securement systems and sutures. We hypothesized that the novel securement systems would be at least non-inferior to all of the catheter securement systems tested.

## Methods

### Materials

The following securement systems were applied to the inserted catheters per randomization schedule and manufacturer’s application instructions. The samples were allowed to dwell on the skin for 10 min prior to testing.

Test materials (commercially available at the time of the experiments):

- Securement device: StatLock® stabilization device (Bard PICC Plus Stabilization Device, Item #VPPCSP, Bard Medical, Covington, GA, USA), covered with a bordered IV cover dressing (3M™ Tegaderm™ Transparent Film Dressing with Border cat.1655, 3M Company, St. Paul, MN, USA).
- Securement dressing: SorbaView® SHIELD dressing (Cat. # SV353 UDT, Centurion, Williamston, MI, USA).
- Sutures: 3–0 silk suture (Ethicon, Somerville, NJ, USA), covered with a bordered IV cover dressing (3M™ Tegaderm™ Transparent Film Dressing with Border cat.1655, 3M Company, St. Paul, MN, USA).

Investigational materials

- Investigational device 1: 3M™ PICC/CVC Securement device + 3M™ Tegaderm™ IV Advanced Securement Dressing (Cat. #1837-2100, 3M Company, St. Paul, MN, USA): a catheter securement device coated with a silicone adhesive and a plastic retainer used to stabilize the catheter lumens, covered with a bordered dressing. This product was under development at the time of this experimentation and is now commercially available.
- Investigational device 2: 3M™ PICC/CVC Securement device + 3M™ Tegaderm™ CHG Chlorhexidine Gluconate IV Securement Dressing (Cat. # 1877-2100, 3M Company, St. Paul, MN, USA): identical to device 1 but covered with a bordered dressing containing a chlorhexidine gluconate (CHG) gel pad.

### Animal model and catheter insertion technique

This study was approved by the contract lab (Synchrony Labs) institutional animal care and use committee and animal care complied with the *Guide for the Care and Use of Laboratory Animals* and the Animal Welfare Act (9CFR). A pig model was developed by adapting a validated internal test method involving un-inserted catheters placed on the back of humans. This method was modified for use on the pig to include insertion of catheters into the epigastric veins. The investigators were first trained on the validated method prior to proceeding with the animal experiments described below. Training entailed generating peak axial pull force measurements of cut (or un-inserted) catheters placed on the back of a pig using four out of the five study materials (sutures excluded).

### Pig model

The study used 26 large female farm pigs (48 ± 3 kg) obtained from Palmetto Research Swine (Reevesville, SC, USA) and was completed in the surgical suite of a facility specializing in pre-clinical models (Synchrony Labs, Durham, NC, USA). The pigs were sedated with Telazol® (4–6 mg/kg) prior to the procedure, and general anesthesia was induced using 5 % isoflurane dispensed through a face mask and maintained with 2–2.5 % isoflurane via endotracheal tube. Animals were placed on the surgery table in dorsal recumbence. Standard ECG leads were applied, and a venous catheter was placed for infusion of fluids in the ear, a location away from the test sites. The animals were intubated, anesthetized, and ventilated prior to testing and were prepared for aseptic abdominal surgery using a standard procedure. The hair on the abdomen was removed using an animal clipper with a #40 blade, and the area was washed with water only. The abdomen was then wiped with 70 % isopropyl alcohol (IPA). Each insertion site on the abdomen was scrubbed with a 10.5 ml, clear ChloraPrep® Patient Preoperative Skin Preparation (CareFusion, San Diego, CA, USA), a 2 % chlorhexidine gluconate/70 % isopropyl alcohol formulation. The ChloraPrep® was allowed to air-dry for at least 5 min to ensure optimal adhesion of the products to skin. In the case of the securement device, the skin was further prepped with the skin protectant included with the device and allowed to dry. During the procedure, animals were monitored for the presence of spontaneous movement and blood oxygen saturation and if needed, appropriate adjustments to anesthesia were made. At the end of the procedure, the animals were given a dose of 40 mEq IV of potassium chloride (KCl) and removed from the ventilator following the American Veterinary Medical Association guidelines on euthanasia [12].

### Catheter insertion

A triple lumen 7 Fr., 16-cm-long Arrow®-Howe’s Multi-Lumen Central Venous Catheter (Teleflex® Inc., Research Triangle Park, NC, USA) was aseptically inserted into one of four insertion sites of the superficial epigastric veins and spaced to allow one catheter per location in the right and left upper abdomen and right and left lower abdomen. Insertions were performed using a longitudinal “cut-down” technique to surgically expose the epigastric vein and insert the catheter into the vein under direct vision. The catheter tip was directed toward the animal’s head so that the catheter lumens could be directed caudally.

**Fig. 1:**
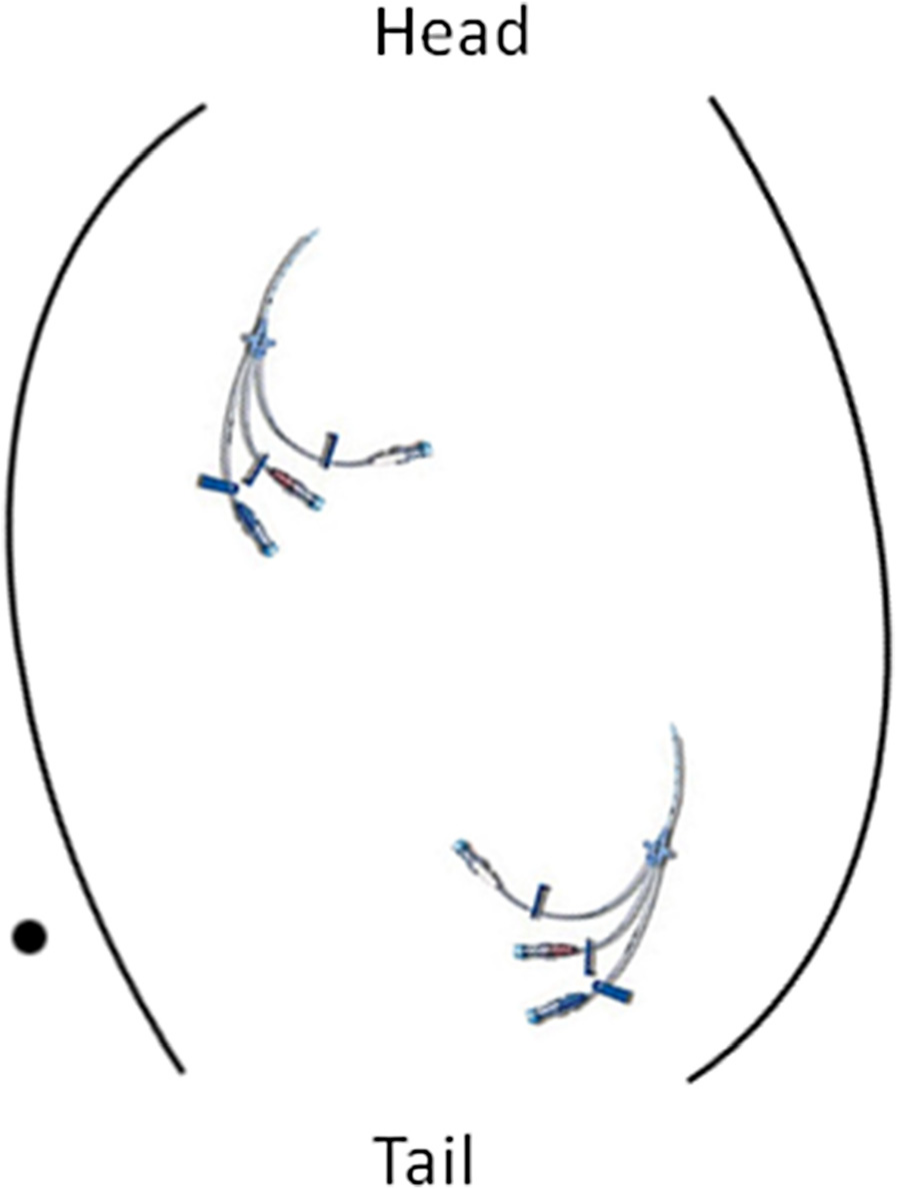
Schematic view of the pig abdomen with two catheters inserted (*right* cranial, *left* caudal)

In order to provide enough space between securement systems, only two catheters were inserted at one time and placed diagonally from one another, e.g., right cranial and left caudal aspects of the abdomen as illustrated in Fig. 1. The skin where each catheter exited was marked with an indelible skin marker. After the first set of two catheters and securement systems were tested, they were pulled out and the second set of two catheters and securement systems were positioned and tested (left cranial, right caudal).

### Application of the various catheter securement systems

**Fig. 2:**
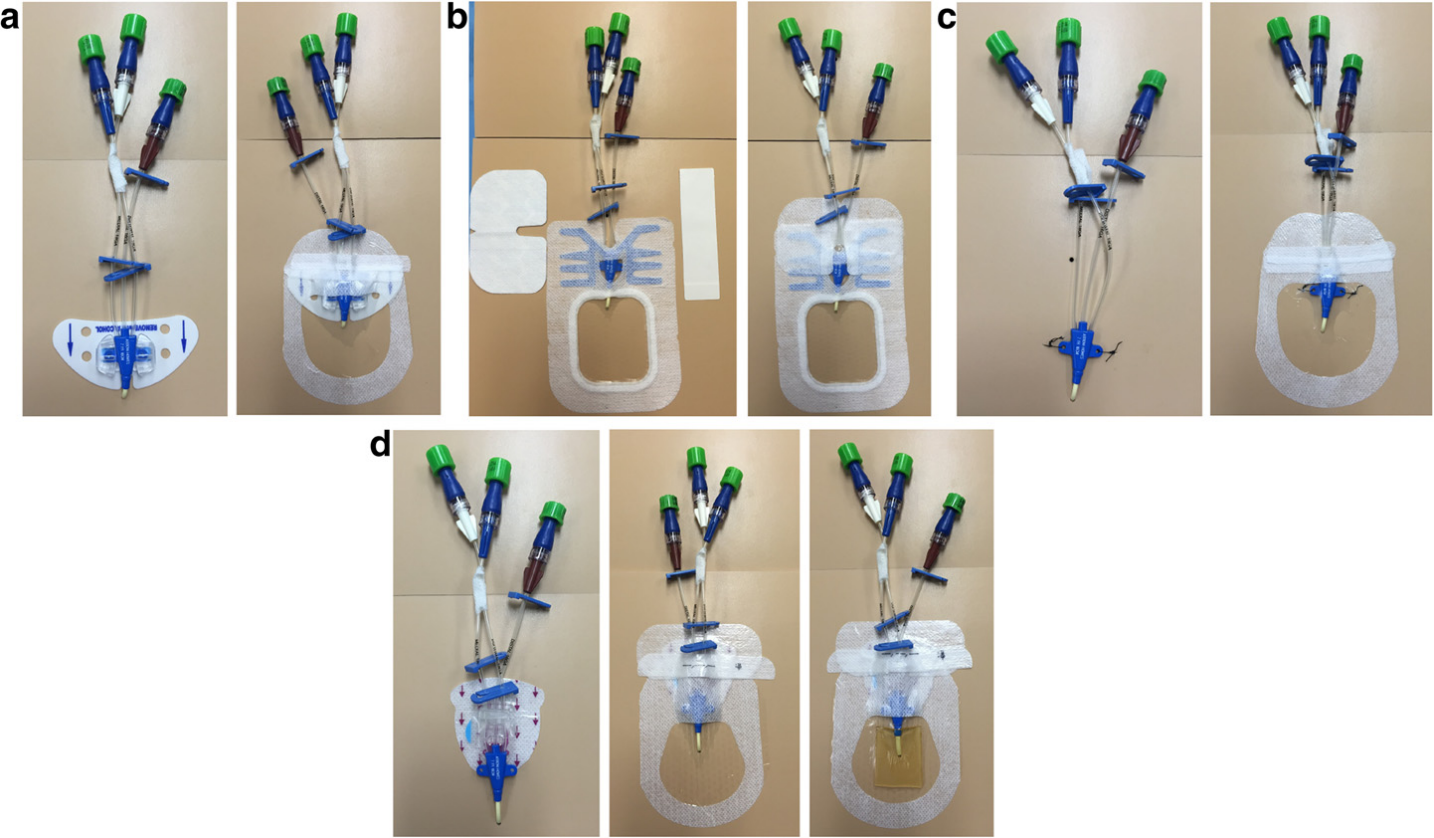
Application of the investigational materials. **a** Securement device. **b** Securement dressing. **c** Sutures. **d** Investigational devices 1 and 2. For each device, the first photo shows the fixation device itself, and the second photo shows the final result after the device is covered with the appropriate dressing. For the investigational devices, the first photo shows the fixation device itself, the second photo shows it covered with the dressing without the CHG pad, and the third photo shows the fixation device covered with the CHG dressing

Figure 2 provides illustrations of the various devices and how they were applied, as modeled on a flat surface for better photographic results. For each device, the first photograph shows the fixation device itself and the second photograph illustrates the device covered with the appropriate dressing. Each device that was not a dressing was covered to comply with its instructions for use to ensure the most clinically relevant test result. For the investigational devices, the first photo shows the fixation device itself, the second photo shows it covered with the dressing without the CHG pad, and the third photo shows the fixation device covered with the CHG dressing.

For the securement device (Fig. 2a) and the securement dressing (Fig. 2b), the manufacturer’s instructions were followed. The securement device was covered by a bordered film dressing (3M™ Tegaderm™ Transparent Film Dressing with Border).

Suturing technique (Fig. 2c): To anchor the catheter to the skin, a 3–0 silk suture on a curved needle was first passed through approximately 5-10 mm of skin under each of the two catheter eyelets. After a square knot was tied, the suture was then threaded through each catheter eyelet and three interlocking square knots were made using a needle driver. A dressing was used to cover the insertion site and sutures.

For the investigational systems (Fig. 2d), which consisted of both a device and a dressing, the following application method was used.

First photo: The device was oriented with arrows pointing toward the insertion site. The catheter was placed into the device, and the lumens were woven under the single plastic arm. A large, notched, film-covered soft cloth tape strip is included in the system. The liner of the attached tape strip was removed, and the lumens were secured to the device base by the tape strip. The device was positioned on the skin at the desired location, and the liners were sequentially removed. Pressure was applied to the device base to establish good adhesion to the skin.

Second photo (investigational device 1): After application of the device, 3M™ Tegaderm™ IV Advanced Securement dressing was applied by peeling the liner and placing the dressing so the transparent film covered the insertion site and the border of the dressing covered the single plastic arm on the device. Firm pressure was applied on the dressing including edges to enhance adhesion to the skin. The frame was removed slowly while smoothing down the dressing edges and pressing from the center toward the edges to enhance adhesion. The sterile tape strip was then applied by removing the liner, grasping the non-adhesive tab, and bending slightly with the thumb. The catheter lumens were lifted and the notch end of the tape strip was applied under the lumens and over the dressing edge. Pressure was applied on the tape strip to enhance adhesion. The frame was then removed from the tape strip.

Third photo (investigational device 2): The dressing used to cover the device is 3M™ Tegaderm™ CHG Chlorhexidine Gluconate IV Securement Dressing.

### Catheter preparation for testing

**Fig. 3:**
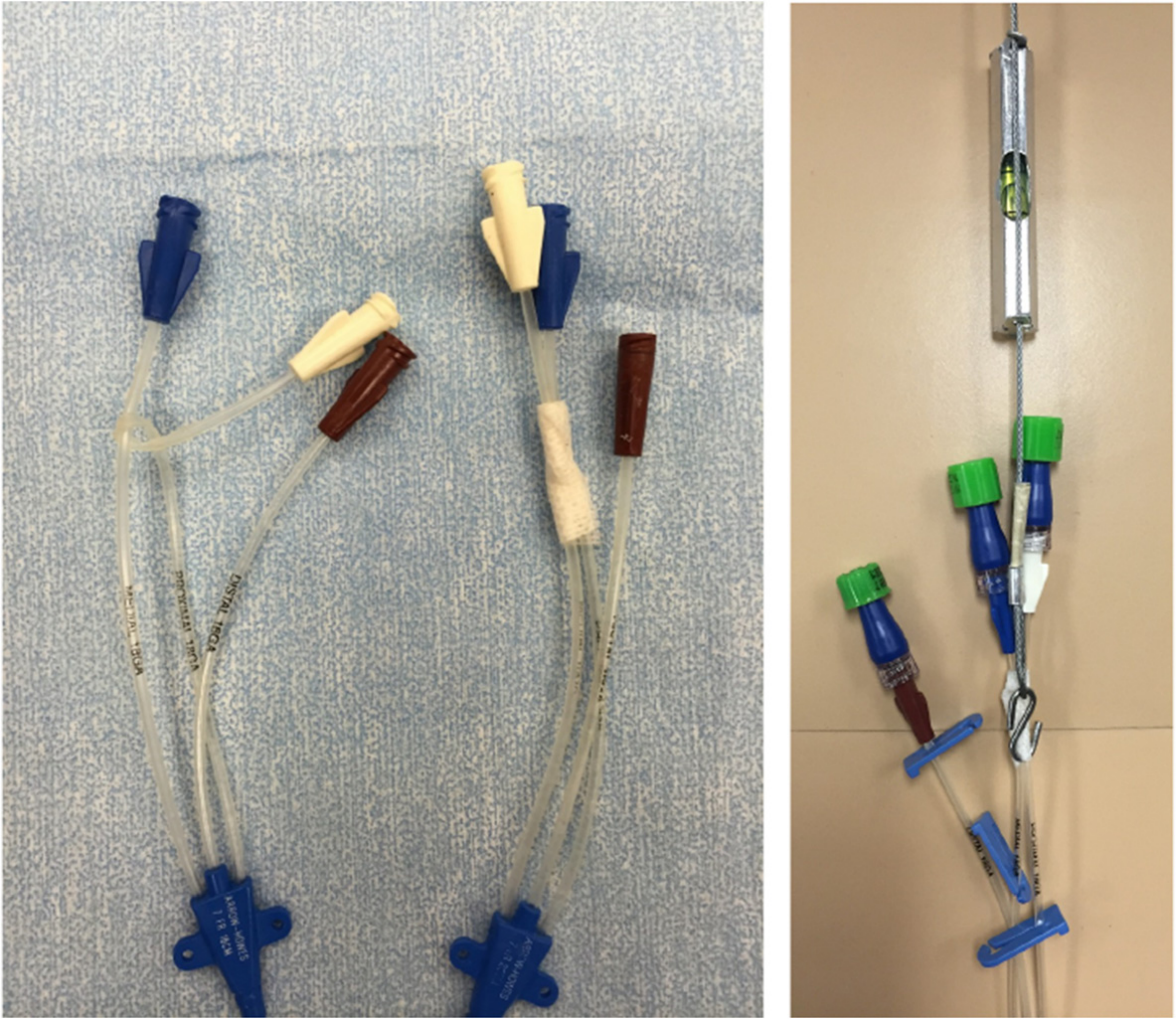
Lumens tied in a knot and taped; IMASS hook placement on lumens for pull test

To attach the catheter to the IMASS pull tester, the three lumens were clamped and a loop was created by tying the outer two lumens together in a half knot at their distal ends. To prevent the knot from unraveling, a narrow strip of tape was applied to encircle the knot. The IMASS machine was positioned at the foot of the O.R. table, and the “s” hook on the end of the IMASS connection cable was attached to the catheter loop created earlier. This is illustrated in Fig. 3. Using a bubble level attached to the IMASS connection wire, the height of the IMASS table was adjusted so that the IMASS connection wire was axial (level) to the catheter insertion site from which the catheter was pulled.

### Catheter securement testing

The peak axial pull force test was used to measure catheter securement. In this study, catheter securement was defined as the peak force (*N*) obtained when either of the following failure events occurred (whichever came first):

- The catheter moves 1 cm or greater out of the insertion site (this amount of motion is clinically relevant as catheter tip migration from the cavo-atrial junction can lead to significant complications in a clinical setting [9])
- The dressing and/or device lifts off the skin
- The catheter pulls out from under the dressing
- The doors of the securement device open
- The sutures break

**Fig. 4:**
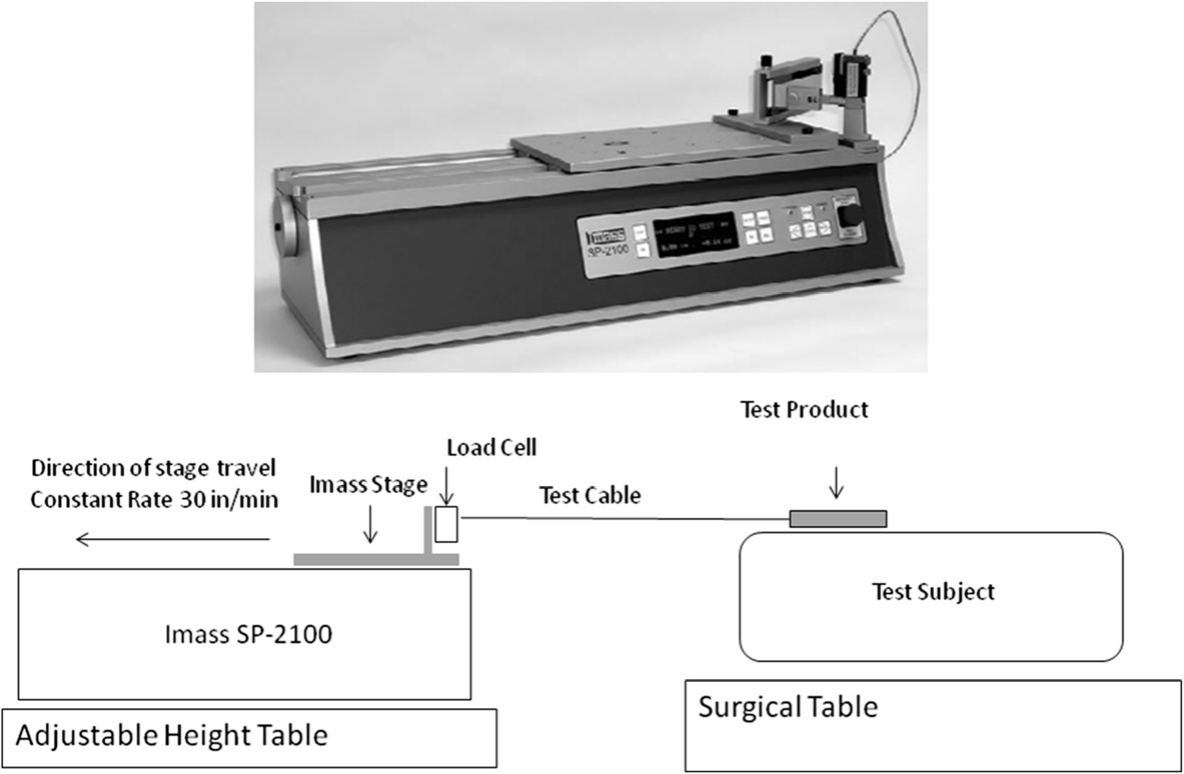
Peak axial pull force test equipment

The peak axial pull force was measured using an IMASS Model SP-2100 Slip/Peel Tester (IMASS Inc, Accord, MA, USE). The raw peak axial pull force was collected in the units of gram-force, which was converted to the SI unit of Newton using the following formula: 1 g-force = 0.00980665 Newton (N). For comparison, a force of 40 N corresponds to 9 lbs (4.82 kg) of force. The system is described in Fig. 4. Ten minutes after the application of the securement system, the video recording of the entire pull force profile was started, and the pull force (tensile distraction force) was applied at the rate of 76.2 cm/min (30 in./min). The peak axial pull force measurement was obtained when securement system failure occurred. The peak axial pull force (securement of the catheters) was recorded for each securement system applied to each pig. Video recording of the entire pull force profile was obtained and later reviewed. Once peak axial pull force testing was completed with the first two catheters, those were removed, manual pressure was applied to the sites to control bleeding, and the other two insertion sites were accessed. The peak axial pull test method was then repeated for the other two catheters.

### Statistical analysis

A sample size of 25 (no replicates per product on the pig) was planned in this study based on a power calculation. This sample size would achieve at least 80 % power to detect a difference of 454 gram force or 4.45 N (using an adjusted Tukey test) if the within-pig standard deviation (SD) was at most 420 gram force (4.12 N), assuming 94 % efficiency with the incomplete block design of having five samples to choose from with a maximum of four samples per pig.

Peak axial pull force analysis was conducted using a mixed effects model at significance level of 0.05 (two-sided). Sample and sample location (upper versus lower sites) were used as the fixed effects in the model, while subject (pig identifier) was included in the model as the random effect. The sample-by-location interaction term was tested for significance. Heterogeneity was modeled in which each sample was allowed a different covariance parameter.

Non-inferiority/superiority testing was carried out using the two-sided 95 % adjusted confidence limits and the margin of 4.45 N or 1.0 lb force or 454 gram force. Tukey’s method was used to adjust for multiple pairwise comparisons, which were based on the differences in least squares means.

## Results

### Reproducibility of model

Protocol deviations with respect to sample placement occurred with two early pigs such that there were only three products placed instead of the expected eight products. An additional pig was utilized to obtain four out of the five missing products. This created an imbalance in the placement of sutures and the investigational device 1 samples. Table 1 displays the number of replicates and location for each system tested. The 26 pigs provided a total of 99 sites. The overall sample size consisted of 20 data points for all samples except sutures in which 19 data points were recorded. Table 2 provides the summary of the data collected for each device tested along with information on the reproducibility of this test method.

**Table 1:** Number of replicates for each securement device and location on pig

	Investigational device 1	Investigational device 2	Sutures	Securement device	Securement dressing
Upper right sites	6	5	5	5	5
Upper left sites	5	5	4	5	5
Lower right sites	4	5	5	5	5
Lower left sites	5	5	5	5	5
All sites	20	20	19	20	20

**Table 2:** Summary statistics of peak axial pull force dataset

Sample	Peak axial pull force (Newton)					
	*N*	Mean	SD	Median	Min	Max
Investigational device 1	20	40.41	6.976	40.61	28.42	50.71
Investigational device 2	20	41.01	6.548	40.55	31.99	57.32
Sutures	19	27.56	6.443	26.27	19.06	45.21
Securement device	20	36.98	6.833	37.57	23.70	47.29
Securement dressing	20	17.44	5.453	16.18	10.96	30.73

The sample-by-location interaction term was non-significant. The final model chosen included just the main effects of sample and location (upper vs lower insertion sites). Based on the final model, the within-pig SD was estimated at 0.99 N and the between-pig SD was estimated between 5.16 and 6.91 N, with the securement dressing having the lowest SD and investigational device 1 having the highest SD.

### Performance of the securement systems

**Fig. 5:**
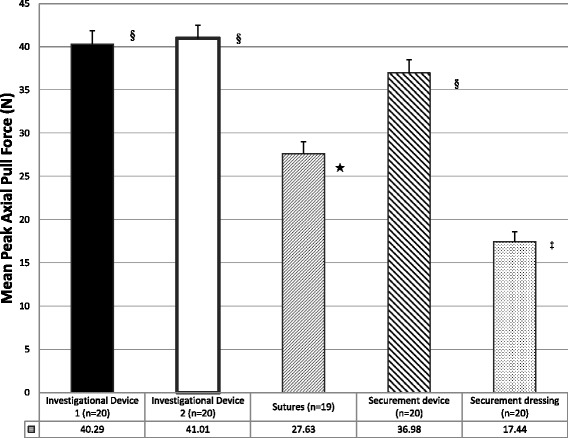
Mean peak axial pull force for each type of securement device (least squares, mean ± standard error). Samples labeled with the *same symbols* are not significantly different from each other. Least squares means differ slightly from the unadjusted means displayed in Table 2 due to the overall imbalance of securement devices placed on pigs

All systems had the same mode of failure, which was the movement of the catheter 1 cm or greater out of the insertion site (catheter dislodgement). In one case, a suture broke on one side of the catheter. Figure 5 illustrates graphically the peak axial pull forces for all methods of securement compared in this study.

Results showed that the two investigational devices displayed the highest mean peak axial pull forces (40–41 N) and were significantly better than sutures (28 N, *p* < 0.0001) and the securement dressing (17 N, *p* < 0.0001) and non-inferior to the securement device (37 N) in this test (Fig. 5). Both investigational systems 1 and 2 were estimated at 3.31 N (95 % CI −1.14, +7.76 N) and 4.03 N (95 % CI −0.28, +8.34 N) higher than the securement device, respectively, and proved to be non-inferior to it based on the lower 95 % confidence limit falling within the non-inferior margin (≥4.452 N).

The securement device required a higher mean peak axial pull force than sutures (*p* = 0.0007) and the securement dressing (*p* < 0.0001) for failure to occur. Finally, the difference in mean peak axial pull force between sutures and the securement dressing also reached statistical significance (28 versus 17 N, *p* < 0.0001).

There was no statistical difference between the two investigational devices, which on average differed from each other by less than 1 N (95 % confidence limits −5.05 N, +3.62 N). Both investigational devices had means that were 12.7–13.4 N higher than sutures and 22.9–23.6 N higher than the securement dressing.

Overall, both investigational devices were significantly better than sutures (50 % stronger), significantly better than the securement dressing (140 % stronger), and non-inferior to the securement device in this test method. None of these devices caused damage to the skin of the animals. However, since the animals were under anesthesia during the whole procedure, the catheter pull test was performed shortly after the application of the securement devices and not several days later to simulate clinical wear time, which is a limitation of this model.

## Discussion

Our results showed that the two investigational devices displayed the highest mean peak axial pull forces (40–41 N) and were significantly better than sutures (28 N, *p* < 0.0001) and the securement dressing (17 N, *p* < 0.0001) and non-inferior to the securement device (37 N) in this test.

A growing body of clinical literature has described the use of securement (or stabilization) devices and dressings for intravenous catheters in order to reduce complications. From this literature, StatLock® appears to be the most commonly studied sutureless catheter securement device for CVCs [13–21]; other devices such as the Nexiva™ Closed IV Catheter System with the customized 3M™ Tegaderm™ IV Advanced Securement Dressing, the Sorbaview® dressings, the HubGuard® catheter securement dressings, and the SecurAcath® device have also been the topic of research publications [22–26]. Catheter securement reduces phlebitis, infection, catheter migration, and dislodgement (reviewed in [13]). Other authors have published papers stating that securement devices help to reduce catheter-related bloodstream infections (CR-BSIs) and suture-related needlesticks and improve cost-effectiveness in patients with central venous access devices, including peripherally inserted central catheters (PICCs) [7, 11]. The investigational devices we describe here have not been tested for CR-BSI reduction at this time. The effect of securement on catheter complications has been reviewed from a clinical standpoint in [27]. These important clinical studies look at factors such as complication rates, ease of use, dwell time, patient comfort, and cost but do not address the mechanical properties such as pull force to compare the various products since it would be unethical to run such experiments on patients.

Although catheter securement systems have been observed to be very useful in clinical use, the rigorous, quantitative testing of their mechanical performance is still in its infancy. Since it would be unethical to subject patients to the risks of tissue injury and catheter dislodgement, models have been created for this type of testing, and they each have their own limitations. For example, forces required to pull out catheters secured with various tapes and taping methods have been measured with a force transducer using a simulated forearm model consisting in PVC pipe [28]. The same group also conducted experiments on the forearm of human volunteers, with catheters taped to the skin but not inserted into veins. We have also used a similar method to test peak axial pull force on human volunteers, in addition to performing a drop test to simulate a fast pull [29]. Other researchers have used pig skin in vitro to suture a catheter adjacent to the catheter entry site and suspend a 1 lb weight to check the tethering, but they have not specified the duration of weight application and observations [30]. In a different study, *postmortem* newborn pig hind legs were used to measure the pull force of catheters secured in different ways (standard or bordered polyurethane films, StatLock®, or tissue adhesives) [31]. It was not specified however whether the catheters were inserted in a vein or just in the tissue. They obtained a mean pull force of about 22 N for StatLock® in their model, versus a mean value of about 37 N using our model, indicating differences between the models. They did not use sutures as a comparator. A porcine hind limb model was also used to compare catheterization techniques for peripheral nerve block and the resulting force required to withdraw catheters, but no securement systems were tested in that study [32]. A different group used the proximal end of discarded extremities after below-the-knee amputations to serve as a surrogate tissue for catheter fixation [33]. In this study, a rod was used to tunnel the catheter under the skin to simulate placement into a central vessel. They compared sutures to two types of staples and measured pull forces. The mean peak force required to dislodge sutures was around 41 N (we were around 27 N in our model). Their study did not test adhesive securement systems.

We have used our animal model to compare various adhesive systems to sutures in the same set of experiments. We chose the pig because of the feasibility of inserting catheters in blood vessels of a size comparable to those of humans and because pig skin has been considered the most similar to humans among the non-primate animals for decades in *in vivo* wound healing research [34, 35]. In addition, we believe that the reaction of pig skin to adhesives is likely to model human skin because both species have a similar total turnover time for the epidermis (30 days for the pig and 26–27 days for humans) [36]. We believe that our model presents an advantage over the other models from the literature, because the catheters were truly inserted into veins of living (anesthetized) animals. The peak axial pull force values measured are therefore likely to better approximate the clinical situation and provide a good basis for the comparison of various securement devices. Since we also generated internal data using the same pull force testing equipment on human volunteers (without insertion of the catheters into veins and without the suture comparison), we are able to comment on the two models. When comparing the pull force data generated in this pig model to the pull force data generated in the human in vivo model, the values were higher in the pig model than in the human model (the difference ranged on average from 4.6 to 9.2 N). Factors related to a catheter being inserted in the veins and the skin of the pig (no moisture or sweating compared to humans) may be contributing to the observed differences in readings. As found in the pig study, the findings in our human study had indicated that the securement dressing required significantly less force to dislodge the catheter than all three of the sutureless securement systems tested, and the three sutureless securement devices showed pull forces that were not different from each other at every time point tested (5 min, 4, and 7 days; manuscript in preparation). Therefore, the comparative performance of the adhesive securement systems tested was the same in both models, confirming the relevance of the pig model in effectively predicting the differences and similarities when measuring peak axial pull force.

This study has some limitations. Since we used a model with animals under anesthesia, the catheter pull test was performed shortly after the application of the securement devices and not several days later to simulate clinical wear time. We also did not torque the catheters or wet the dressings or compare different time points after application. We chose to insert the catheters in epigastric vessels to reduce the number of animals needed compared to using the jugular veins only. We do believe however that our method is informative to compare various devices for their securement performance after application. We chose the Bard StatLock® PICC Plus Stabilization Device as a comparator because we had also used it in our human model without vein insertion and already had comparative data. Moreover, this application is not contraindicated in the instructions for use of this product. We believe our results are relevant for both PICCs and CVCs since our model focuses on the performance of the device when resisting a pull force. For our suture comparator, we selected one type of suture and one fixation method, which may limit the generalizability of the comparison. The possible disadvantages of the new systems are comparable to those of other adhesive securement systems, such as pain or catheter migration while removing or replacing the dressing, and possible skin irritation due to the adhesive on patients with sensitive skin.

## Conclusions

In conclusion, the investigational systems were significantly better than sutures and the securement dressing and non-inferior to the securement device in this pig model using a pull force test method performed 10 min after application of the devices. Securement devices specifically designed to house the catheter performed better than a dressing alone, and such devices also performed better than sutures. Clinically, they offer the advantage of avoiding additional skin perforations for patients as well as the risk of needlestick injuries for health-care workers. The investigational sutureless catheter securement systems described here will provide clinicians with viable securement alternatives to sutures and other sutureless catheter securement systems, while offering the advantage of a convenient all-in-one system. Additional clinical benefits have been identified in a clinical user survey study (manuscript in preparation).

The development of test methods that provide evidence not readily obtained in the clinical setting is important to understand the fundamental mechanical properties of these systems and to quantitatively compare their performance. Clinical studies designed to compare the adverse events observed with various sutureless catheter securement systems will be needed to identify the clinically relevant differences between these securement systems.

## Abbreviations

CFR: Code of Federal Regulations
CHG: chlorhexidine gluconate
CVC: central venous catheter
ECG: electrocardiogram
IPA: isopropyl alcohol
N: Newtons
PICC: peripherally inserted central catheter
PVC: polyvinyl chloride
SD: standard deviation
